# *Legionella pneumophila*—Virulence Factors and the Possibility of Infection in Dental Practice

**DOI:** 10.3390/microorganisms10020255

**Published:** 2022-01-24

**Authors:** Jasminka Talapko, Erwin Frauenheim, Martina Juzbašić, Matej Tomas, Suzana Matić, Melita Jukić, Marija Samardžić, Ivana Škrlec

**Affiliations:** 1 Faculty of Dental Medicine and Health, Josip Juraj Strossmayer University of Osijek, HR-31000 Osijek, Croatia; jtalapko@fdmz.hr (J.T.); erwin.frauenheim@gmail.com (E.F.); martina.juzbasic@fdmz.hr (M.J.); matej.tomas@fdmz.hr (M.T.); suzimatic72@gmail.com (S.M.); mjuki17@gmail.com (M.J.); marija.t.samardzic@gmail.com (M.S.); 2 Faculty of Medicine, Josip Juraj Strossmayer University of Osijek, Josipa Huttlera 4, HR-31000 Osijek, Croatia; 3 General Hospital Vukovar, Županijska 35, HR-32000 Vukovar, Croatia

**Keywords:** aerosol, dental medicine, infection, *Legionella*

## Abstract

*Legionella pneumophila* is defined as a bacterium that can cause severe pneumonia. It is found in the natural environment and in water, and is often found in water tanks. It can be an integral part of biofilms in nature, and the protozoa in which it can live provide it with food and protect it from harmful influences; therefore, it has the ability to move into a sustainable but uncultured state (VBNC). *L. pneumophila* has been shown to cause infections in dental practices. The most common transmission route is aerosol generated in dental office water systems, which can negatively affect patients and healthcare professionals. The most common way of becoming infected with *L. pneumophila* in a dental office is through water from dental instruments, and the dental unit. In addition to these bacteria, patients and the dental team may be exposed to other harmful bacteria and viruses. Therefore, it is vital that the dental team regularly maintains and decontaminates the dental unit, and sterilizes all accessories that come with it. In addition, regular water control in dental offices is necessary.

## 1. Introduction

Members of the *Legionellaceae* family are small, Gram-negative, aerobic bacilli that do not form spores, are capsule-free, and possess the enzymes catalase and oxidase (Table 1) [1]. Amino acids are their primary carbon and energy sources for bacterial growth in the intracellular environment [2]. Amino acids are catabolized by the Krebs cycle, and gluconeogenetic enzymes synthesize sugars from the Embden–Meyerhof–Parnas pathway. *Legionella* is non-saccharolytic and possesses the enzyme protease. The guanine and cytosine content in its DNA ranges from 38% to 52% [3].

Of the known 15 serogroups of *Legionella pneumophila* (*L. pneumophila*), serogroup 1 is present in 84% of cases worldwide (Table 2) [4,5,6]. Serogroups 2 to 15 account for 16 to 20% of *Legionella* pneumonia cases [7]. Patients with *L. pneumophila* of serogroups 2 to 15 showed typical symptoms of *Legionella* pneumonia, although *Legionella* urinary antigen detection tests were negative [8,9,10]. In addition, specific differences in the virulence of different serogroups were observed; for instance, Buse et al. showed a significant difference in the mobility of *L. pneumophila* between serogroups [11]. A characteristic of the *L. pneumophila* genome is the presence of many different eukaryotic-like proteins and protein domains that are probably acquired by horizontal gene transfer [12,13,14].

**Table 1:** Classification of *Legionella pneumophila*.

Characteristics	*Legionella pneumophila*	References
Family	*Legionellaceae*	[15]
Form	bacillus	[1]
Coloring per gram	Gram (-)	[1]
Metabolism	Aerobic	[1]
pH	5–8.5	[16]
Habitat	Aquatic habitats (biofilm, within multicellular organisms)	[17]
Reproduction temperature	25–37 °C	[18]
Survival temperature	0–63 °C	[16]
Nutrients	Amino acids (L-cysteine), iron	[17]
Sensitivity	Drying, chlorine, UV radiation	[19]

**Figure 1:**
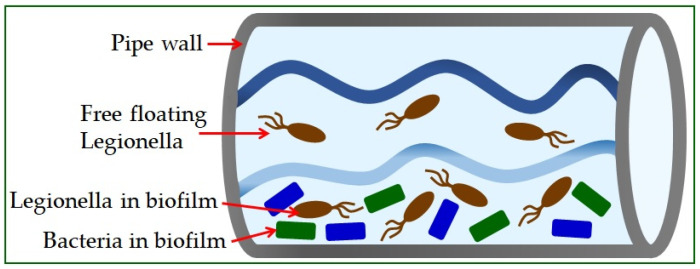
*Legionella* in water supply systems.

*Legionella* is found in the natural or artificial aquatic environment [22]. It occurs in planktonic form or as part of biofilms [23] and has the ability to move into a sustainable but uncultured state (VBNC) [19]. Elevated temperature, inorganic and organic water content, and the presence of protozoa play a crucial role in their growth and spread [24]. The most significant number of *Legionella* is found in water samples with temperatures from 30 °C to 40 °C [25]. Infections in humans occur exclusively by inhalation of contaminated aerosols, which can occur in air conditioning systems, cooling towers, spas, fountains, ice machines, plant sprayers, dental appliances, and showerheads [26]. The material from which the pipeline system is built significantly influences the appearance of high concentrations of bacteria (Figure 1) [5]. The use of copper as a plumbing material has been shown to help reduce the risk of Legionnaires’ disease, while plastic materials support many *L. pneumophila* bacteria [27].

*Legionella* can be found in water distribution cooling towers, where it can replicate within protozoa. It is often *Vermamoeba vermiformis* that protects *Legionella pneumophila* from the effects of heat and disinfectants, which can result in nosocomial infections [28]. Coevolution with multiple protozoan species has resulted in the development of mechanisms that allow *L. pneumophila* to occupy different hosts, and the possibility of human cell infection [2,29,30].

*Legionella pneumophila* is responsible for most cases of legionellosis and is one of the major causes of community-acquired and nosocomial-acquired atypical cases of pneumonia, with a mortality rate between 7% and 25% [20,31]. In comparison, the mortality rate of non-*Legionella pneumophila* species is 5% [31]. Legionnaires’ disease is a pulmonary form of legionellosis with an incubation period of two to fourteen days, and involves severe pneumonia and systemic infection [32,33]. A benign flu-like condition is called Pontiac fever [34]. It is a non-pneumonic disease with unclear pathogenesis requiring no antimicrobial treatment [32]. The mortality rate of adequately treated patients with Legionnaires’ disease varies from 7% to 24%, with immunocompromised and elderly patients being the most susceptible [34]. It is estimated that 25,000 to 100,000 people are diagnosed with legionellosis each year in the United States [35]. In North America and Western Europe, 1–13% of all types of pneumonia were associated with this pathogen [36].

## 2. History of Legionellosis

The two principal clinical forms of bacterial infection caused by inhalation or aspiration of the genus *Legionella*, together with the aerosol in which they are contained, are Pontiac fever and Legionnaires’ disease [26]. Infections obtain their names from the events after which they were described. The identification of Legionnaires’ disease was preceded by a conference of American veterans from the Second World War, the so-called Legionnaire, in 1976, which, as it turned out, became the source of a previously unknown disease [37]; the tragedy was blamed on the ventilation and air conditioning system of a luxury hotel in Philadelphia. Participants of the gathering fell ill and suffered from pneumonia, and there were also fatalities. After this event, the causative agent was isolated in 1977 and named *Legionella* [38,39]. Now, it is known that the 1974 event from the same hotel is also linked to a disease that broke out two years later [40].

This type of bacterium was also responsible for infecting employees and patients of the Health Department in Pontiac, Michigan, in 1968. Although respiratory symptoms were present in both cases, the disease here was milder, with no pneumonia or death; it was called Pontiac fever. A weaker form of the disease in Pontiac could indicate a low infectious dose, but the infection rate among healthcare workers was 95%. This high infection rate suggested that the pathogen was spread through the air [3,37,41].

Among the first discoveries related to *Legionella* spp. was an isolate in 1947 that was described as a “rickettsia-like” organism, and in 1977, it was identified as the same bacterial species and serogroup responsible for the disease in Philadelphia [39,42]. Outbreaks of this disease attract much attention, but it still occurs mainly in individual cases. Its occurrence has been reported in Europe, the USA, Canada, New Zealand, Japan, Singapore, and Australia. The registered case number of infections is increasing despite numerous guidelines on preventing the spread of *Legionella* spp. [43]. Today, over 60 species of *Legionella* are known. *Legionella pneumophila* is the most common pathogenic species and includes 15 serogroups, although most human diseases are caused by the *L. pneumophila* serogroup 1 [44]. Other species that are clinically significant for human infection, in addition to those shown in Table 2, are *Legionella feeleii*, *Legionella micdadei*, *Legionella longbeachae*, *Legionella anisa*, *Legionella dumoffii*, and *Legionella bozemanii* [21,45,46].

## 3. Virulence Factors

The development and clinical form of the disease depend on the number of bacteria in the aerosol, the serogroup to which they belong, the virulence factors, and the person’s immunity. Therefore, in addition to preventing the colonization of water bodies, it is also essential to determine the virulence factors of *Legionella* spp. [47]. Initial adhesion to cell surface receptors is associated with the bacterium’s surface structures, namely pili (fimbriae), lipopolysaccharides, and proteins [48]. Potential invasiveness is increased by flagellar motility and toxin production [46]. Microbial pathogenicity enhances the possibility of long-term intracellular survival and replication in alveolar macrophages, thereby increasing infectivity while bypassing the patient’s immune system [49].

### 3.1. Surface Virulence Factors

Surface virulence factors that affect the virulence of *Legionella* spp. and enhance infectivity are lipopolysaccharide (LPS), flagella, pili, and outer membrane proteins [46,47,49,50,51].

The LPS is located on the outer envelope of the outer membrane and is crucial in interacting with various host immune cells, as well as in intracellular traffic modulating [22]. The LPS molecule consists of an O-specific chain, a nucleus, and a lipid A component, combined with an endotoxin with a relatively low toxic potential. LPS contains many extended, branched fatty acids and O- and N-acetyl groups. It is highly hydrophobic and differs from the lipopolysaccharides of other Gram-negative bacteria [51,52].

*Legionella* spp. move, in most cases, by means of a polar and/or lateral flagella consisting of a basal body, a hooked structure, and a filament [53]. Motility may be crucial for dispersing in the lungs of patients, as such forms of *L. pneumophila* have been detected in alveolar parts [46,51,52,54]. The gene responsible for expression depends on the temperature, availability of nutrients, and viscosity of the medium in which they reside. Although flagella are not a condition for intracellular proliferation, they enhance host cell invasion regardless of attachment to it [51,52]. Pili can be divided into two forms: the long form, measuring 0.8 to 1.5 µm, and the short form, measuring 0.1 to 0.6 µm. The PilE protein is an integral part of the long form of type IV pili. It is involved in binding and adhesion to host cells. Prepilin peptidase (PilD) is another protein liable for the production of type IV pili. It is crucial for successful intracellular proliferation. This protein is also involved in secretion type II [51,52].

*Legionella* spp. outer membrane proteins are essential for phagocyte entry and survival. The Macrophage Infectivity Potentiator (MIP) displays peptidyl–prolyl cis/trans isomerase activity and is necessary for the early stages of intracellular infection and survival in macrophages, protozoa, and pulmonary epithelium [55]. Due to the *MIP* gene’s characteristics not being found in other *Legionella* genes, it is possible to identify clinical and environmental strains of *L. pneumophila* and other *Legionella* spp. by *MIP* sequencing [51,56].

The major Outer Membrane Protein (MOMP) has porin properties, and is required for bacterial interaction with CR1 and CR3 receptors on monocytes and other phagocytes [57]. Factors of both the host and bacteria that facilitate initial adherence and entry of *Legionella* spp. into the cell, are essential [57].

### 3.2. Secreted Factors

*Legionella* spp. secretes various pigments, toxins, and enzymes [58]. Moreover, *Legionella* spp. secretes more than 18,000 proteins containing eukaryotic-like domains, called effector proteins, through secretion systems [13]. Many effector proteins are secreted into the host cell, facilitating *Legionella* intracellular replication [13]. These secretion systems are essential virulence factors in *L. pneumophila*.

The type I secretion system known as Lss consists of the ABC transporter (ATP-binding cassette), a membrane-fusion protein, and an outer-membrane protein [59]. The *lss* gene cluster, *lssXYZABD*, which includes the ABC transporter and membrane fusion protein, was found in all *L. pneumophila* strains [50].

Secretion system type II, termed Lsp and often referred to as the general secretory pathway, contains numerous degradation enzymes, including RNase, two acid phosphatases, zinc metalloprotease, etc. [60,61]. The *L. pneumophila* type II secretion system has 12 components. Some components are the prepilin peptidase *pilD*, the outer membrane secretin and ATPase *lspDE*, and the pseudopilins *lspFGHIJK*, *lspC*, and *lspLM* [57]. The Lsp secretion system is essential for *L. pneumophila* survival at low temperatures [50].

The type III secretion system is a protein-transport mechanism that translocates cytoplasmic substrates directly into the host cytoplasm [62]. It contains flagellum-encoding genes [59] and secretins as part of a type II secretion system, homologous to the DotD protein in a type IVB secretion system [63].

Another important secretory system is the type IV secretory system. There are two subclasses of the type IV system—IVA (called Lvh) and IVB (called Dot (defect in organelle trafficking) /Icm (intracellular multiplication)) [59]. The Dot/Icm secretory system is encoded by the *Dot*/*Icm* genes [63]. The Dot/Icm secretion system secretes more than 300 different effector proteins into the host cell and is crucial for the virulence of *L. pneumophila* [13]. Therefore, the Dot/Icm secretion system constitutes around 10% of the *L. pneumophila* proteome, suggesting that the effectors include a significant determinant of *L. pneumophila* survival [50]. The Dot/Icm system is vital for establishing a replicator niche and avoiding lysosomal/endocytic fusion [64]. The action of bacterial degradation enzymes ultimately leads to the death and lysis of host cells and damage to lung tissue [65,66]. In addition, the Dot/Icm effector proteins are required to translocate the *Legionella*-containing vacuole across the membrane [23]. The Lvh secretion system contains genes encoding mobility factors and enzymes [59]. It may have a role during intracellular replication of *L. pneumophila* and thus complement Dot/Icm function [50]. In addition, the Lvh can functionally replace defective Dot/Icm [67].

Type II and IVB secretion systems are found in all *Legionella* strains, while the type I secretion system is exclusive for *L. pneumophila* [65]. In addition, type II and IVA secretion systems are found in some *Legionella* strains [59]. However, some effector proteins of type II, III, and IVA secretion systems are homologous to the components of the type IVB secretion system, and are therefore not found in all *Legionella* strains [63]. Secretory systems are essential for virulence, and *Legionella* species that have additional secretory systems have increased pathogenicity [59].

The brown pigment, or pyomelanin, is one of the frequently studied secreted factors. It is a polymer formed with homogenetic acid (HGA), produced by bacteria with oxygen [68]. The *lly* gene is necessary for its formation [69]. Mutagenesis of *Lly* gene does not affect intracellular replication within amoebae or macrophage-like host cells [69]. Secreted HGA becomes toxic in the presence of oxygen [70]. Pyomelanin protects *Legionella* from possible light-induced damage and helps it to obtain iron, an essential micronutrient for its survival [51,58].

### 3.3. Biofilm

Nowadays, the increased use of drugs (especially antibiotics) has led to bacterial resistance, a significant problem in patient treatment [71]. The biofilm is one of the protective mechanisms that increases the resistance of bacteria to specific external agents [72,73]. *Legionella* spp. is characterized by the ability to survive in a biofilm and achieve high virulence and resistance, even after severe physical and chemical treatments [74]. In addition, many biological factors influence the persistence of the biofilm [75]. As such, the biofilm allows more excellent adhesion of specific bacterial species through various stages, from initiation to maturation of the biofilm and the formation of an extracellular matrix [74,76,77,78]. Survival of *Legionella* spp. under oligotrophic conditions with a lower content of organic matter and nutrients requires the incorporation of *Legionella* into the microbial community with other bacteria [79,80]. On this basis, it is possible to explain the increased numbers of *Legionella* spp. in artificial habitats such as hot water systems [81]. Bacteria in these communities participate in interactions such as the food chain formation or the congregation continuation. The microorganisms in the biofilm may also repel other microbes that are unlikely to contribute to the community [58,80].

For *Legionella* spp., biofilm formation is essential for survival [25]. Like many other microorganisms, it responds to environmental factors that significantly affect biofilm formation and/or colonization. Temperature is an influential agent affecting biofilm colonization and can affect the biofilm determinants produced by *Legionella* spp. In vitro, monotypic biofilms at 37–42 °C consist of filamentous bacteria, but at 25 °C, they are thinner and dominated by rod-shaped bacteria. *Legionella* cell length is associated with ppGpp signaling. A biofilm at 37 °C is much more solid than at 25 °C, and interestingly, at 25 °C, it is more prone to better adhesion potential [29,82]. Specific genes may also regulate biofilm formation, such as the putative twin-arginine translocation pathway involving the *tatB* and *tatC* genes [83]. In the same case, expression of the *MIP* gene has been found to promote biofilm creation at an early stage, when *Legionella* spp. does not require a host for growth [83,84].

Quorum sensing (QS) in Gram-negative bacteria regulates the gene expression of various bacterial processes, including biofilm formation [85]. Bacteria that show QS signaling are usually found in artificial aquatic systems and may regulate biofilm production in the environment [86]. The QS autoinductor used by *Legionella pneumophila* is LAI-1 (3-hydroxypentadecan-4-one), which produces and detects the Lqs system and contains the autoinductive synthase LqsA, homologous sensory kinase LqsS, and the LqsR response regulator [87]. The quorum sensing autoinducer (3-oxo-C12-HSL) possessing *P. aeruginosa* inhibits *L. pneumophila* biofilm formation [88]. This effect is associated with a decrease in LqsR. This suggests that QS could play a role in the dispersion of *L. pneumophila* during the later stages of biofilm development [89]. Several biological factors such as microbial communities, temperature, and specific genes regulate *L. pneumophila* biofilm production. These factors should suppress biofilm formation to reduce colonization in aqueous systems [74].

### 3.4. Legionella and Protozoan Interactions

Certain species of protozoa are crucial for the growth of *Legionella* in natural and artificial environments [90]. Accordingly, the presence of *Legionella* in these environments depends on the spectrum of protozoa present in the host [91]. *Acanthamoeba*, *Hartmannella*, and *Naegleria* are most commonly isolated from *Legionella*-contaminated water systems [92]. Other protozoa associated with *Legionella* are *Saccamoeba*, *Vexillifera*, and *Platyamoeba* [93].

**Figure 2:**
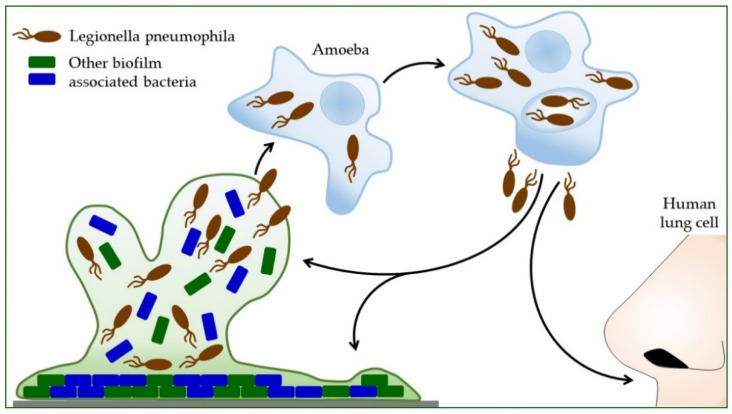
The life cycle of *Legionella pneumophila*. *Legionella* reproduces only inside other cells. Bacterial-feeding amoebae also live in the environment where *Legionella* is found. After the *Legionella* is eaten by the amoeba, it is encapsulated inside the amoeba, where it continues to grow and multiply. By releasing *Legionella* bacteria from the amoeba, they can disperse into the environment and form a new biofilm with other bacteria, or humans can inhale them. In humans, this cycle is repeated, but in this case, the human lung cells are infected.

Propagation within the amoeba *L. pneumophila* increases the ability to produce polysaccharides, which increases its ability to form a biofilm (Figure 2) [74]. Protozoa provide nutrients for intracellular *Legionella* and protect them from adverse environmental influences. Bacteria survive high temperatures, disinfection procedures, and drying inside the *Acanthamoeba* cyst [19]. *L. pneumophila* can use protozoa to colonize new habitats, so inhaled protozoa are an effective mode of transmission to humans [94]. Thus, the symbiosis of *Legionella* and protozoa contributes to the infection process itself [22]. After intracellular replication within the protozoan, *L. pneumophila* shows more excellent resistance to stress [30,95]. During coevolution with protozoan cells, *L. pneumophila* acquires highly sophisticated and diverse strategies for taking over the host cell process [96,97]. It secretes hundreds of effectors into the host cell, controlling host signaling pathways and key cellular processes [57,98,99]. *L. pneumophila* can also alter host transcription and translation processes and utilize epigenetic mechanisms in the cells in which it is found to counteract host responses [100].

Upon internalization, intracellular bacteria reprogram the endosomal–lysosomal pathway of host degradation [101]. The multiplication of *Legionella* bacteria within a maturation-blocked vacuole, which fails to acidify and fuse with lysosomes, shows many similarities to human phagocytic cell infection [102]. It includes the recruitment of the rough endoplasmic reticulum surrounding the membrane-bound vacuole. Interaction with protozoa is thought to be the driving force in the evolution of *Legionella* pathogenicity. In recent years, tremendous progress has been made in unraveling the mechanisms by which intracellular pathogens attack host cells and establish intracellular infections [103].

## 4. *Legionella pneumophila* in Dental Practice

Dental staff may be at high risk of *Legionella* infection, and therefore, an occupational risk assessment is required. In addition to many dentists, other healthcare professionals in the dental clinic, such as dental assistants and hygienists, are also exposed to the occupational risk of *Legionella* infection. It is estimated that the occupational risk of *Legionella* infection may affect 1 to 2 million healthcare professionals worldwide [104].

Based on research in dental offices conducted in 1986 in Austria, the presence of *Legionella pneumophila* serogroup 1 was determined in 10% of water supply systems. The first death of a dentist due to Legionnaires’ disease was in 1995, and *Legionella* was discovered in the plumbing system of his office [104]. In 2012, an 83-year-old patient from Italy died of Legionnaires’ disease, and the source of the infection was contaminated water in the dental office she visited [105]. In addition, in the same year, an elderly, immunocompromised man in Sweden died due to *Legionella* in the cup filler outlet used for rinsing at the dental ward [106]. *Legionella pneumophila* is fatal in many cases [20]. However, the cause of the fatal outcome related to the dental practice was determined only in the patients shown. According to a study by Kevorkyan et al., antibodies to *Legionella* were significantly higher in medical and dental professionals than in non-professionally exposed subjects [107]. The possibility of contamination in a dental unit water system with microorganisms has been discussed since the beginning of dental chair use. Due to the constant exposure of patients and staff in the dental team to the aerosol produced during operations, the microbial quality of the water is critical. Water supply systems might contain opportunistic and pathogenic bacteria, mostly Gram-negative species that pose a particular risk in immunocompromised individuals [108,109]. The water systems of the dental unit can be initially contaminated through water coming into the system or by pulling and sucking saliva and other fluids from the patient’s mouth. Dental chairs can receive water through a public water supply network or special tanks built into the chair to pour liquid. Other factors that might influence this are the work unit’s model and the time of its construction, whether there is a built-in system against the return flow of patient fluids, infection prevention measures, used disinfection methods, etc. [109,110].

The guidelines for the allowable number of bacteria in the dental unit’s water are different and mostly coincide with the number of microorganisms allowed in the drinking water. In Europe, this number is up to 100 colony-forming units per milliliter of water (CFU/mL). The American Dental Association (ADA) set the allowable number of microorganisms in the water for dental supply at ≤200 CFU/mL, while the Centers for Disease Control and Prevention (CDC) recommended that the number be ≤500 CFU/mL [109,110,111,112,113,114]. The risk of infection arises because most instruments that are necessary for work in dentistry, such as micromotors, turbines, sonic and ultrasonic scalers, water/air syringes, etc., produce an aerosol in the inhalation zone. The presence of *Legionella* spp. in saliva and dental plaque biofilm has also been shown [115,116].

**Figure 3:**
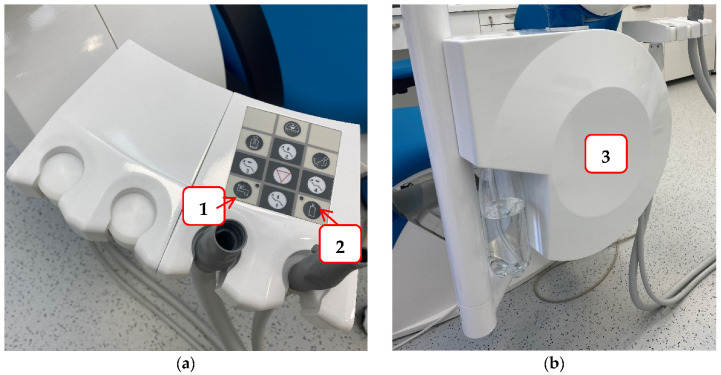
An example of a dental unit with a choice of water supply: Panel (**a**) the buttons that allow the selection of water supply either from the public network (1) or a bottle of distilled water (2); Panel (**b**) water tank for distilled water.

To reduce the possibility of infection and protect staff and patients in the dental office, it is necessary to try to prevent contamination of the hydraulic system of the dental unit. Some of the measures that can be achieved are: a dental unit that can be connected to sterile or distilled water (Figure 3); flushing water through the instruments at the beginning and end of the working day, and also between each patient to prevent cross-contamination and water stagnation; continuous disinfection; procurement of thick filters, etc. [117]. For this reason, dentists are required to conduct a legal risk assessment of their hydraulic systems, identify and assess the sources of risk, and prepare guidelines for the prevention and control of the risk of *Legionella* infection. Furthermore, they must monitor the quality of their hydraulic systems once a year to ensure that the hydraulic systems are free of *Legionella* [117].

Particular attention should be paid to prevention measures in these times of the COVID-19 pandemic. COVID patients are more susceptible to secondary infections for several months during recovery. Lock-down and government measures such as staying at home and delaying the procedures also result in prolonged water standing in the dental unit’s supply tanks. Hence, the biofilm accumulation in the system is more likely [116,118,119].

### 4.1. Resistance of L. pneumophila Biofilms to Biocides

*L. pneumophila* poses a constant threat to human health in anthropogenic water sources [21]. Due to the intracellular lifestyle within protozoa, it is difficult to assess whether the resistance of *L. pneumophila* in environmental biofilms is due to the structure of the biofilm, its association with amoebae, or both [120]. However, the fact is that *L. pneumophila*, which is found in biofilms, is highly resistant to the action of biocides [74].

Numerous disinfection methods were used to limit the growth of *L. pneumophila*, but none succeeded in complete eradication; namely, recolonization occurred very soon after treatment [120,121]. In addition, some studies showed that the biocide action on the *L. pneumophila* biofilm could lead to the transition of the bacterium to the VBNC state [74]. The most common biocides used in *L. pneumophila* water disinfection protocols are chlorine and chlorine derivatives. However, they show efficacy only on planktonic cells, but not on biofilm [122].

Two reasons for this are the resistance of *L. pneumophila* to disinfectants; one is due to its ability to survive within the biofilm, and the other is that it possesses an intra-amoebic lifestyle [29]. Namely, vesicles containing intracellular *L. pneumophila* released by the amoeba are resistant to biocidal treatments. It is important to note that these vesicles remain viable for several months [74]. Chlorine dioxide, unlike chlorine, can penetrate the biofilm and can also inactivate free-living amoebae which *L. pneumophila* inhabits. Therefore, it is concluded that chlorine dioxide can be used as a secondary disinfectant to reduce the risk of Legionnaires’ disease in hospital systems [19].

The use of phages in the treatment of biofilm infections is known in many bacterial pathogens [123], so the addition of specific phages can be used to control the growth of *L. pneumophila.* However, the phage can degrade polysaccharides and destabilize the biofilm [124].

The antimicrobial activity of silver has long been known, and silver is an increasingly frequent target of research to find new antimicrobial agents [125]. When it comes to a significant reduction in the volume of *L. pneumophila* biofilm, silver nanoparticles have shown outstanding results [126].

Natural compounds that have demonstrated antimicrobial efficacy on *Legionella* strains are antimicrobial peptides, biosurfactants, and essential oils [19]. Different filtration methods are possible, but as filters have a certain lifespan, this could significantly increase the cost of maintenance in the hospital system [127].

### 4.2. Antimicrobial Therapy

*Legionella* possesses the enzyme ß-lactamase. Therefore, beta-lactam antibiotics are ineffective in treating legionellosis [128]. For that reason, azithromycin and fluoroquinolones, including levofloxacin and moxifloxacin, are recommended for *Legionella* pneumonia in some guidelines [129,130]. In addition, treatment of legionellosis is long-term, from 7 to 14 days, while the symptoms themselves are not present for too long [131,132].

## 5. Conclusions

In dental offices, along with many other potential causes of infections for both staff and patients, there is a possibility of exposure to the bacterium *Legionella pneumophila*, which can cause severe pneumonia—Legionnaires’ disease. Vulnerable patients are the immunocompromised and elderly, and chronic disease patients such as those with chronic obstructive pulmonary disease, cardiovascular disease, and diabetes. *Legionella* infection could be fatal for patients on hemodialysis and with kidney transplants. Smoking and alcoholism are risk factors for Legionnaires’ disease. The most common reservoir of *Legionella* in dental practices are water tanks, and the route of spread is through contaminated aerosol generated during the use of dental instruments. Understanding the molecular mechanisms responsible for intra-amoebic related resistance is necessary, and would result in the development of new strategies for eradicating *L. pneumophila*. It is essential to know the breeding characteristics of *L. pneumophila*, as well as its virulence factors, and spread methods. Knowing the hygienic measures and that disinfectants can be used to prevent the spread of *L. pneumophila* is imperative. It is necessary to carry out water control, appropriate sampling in dental offices, and microbiological processing of samples.

## Figures and Tables

**Table 2:** *Legionella* species, serogroup details, and their ability to cause human infection and mortality rate [7,17,20,21].

*Legionella* sp.	Serogroups Associated with Human Disease	Diseases	Mortality Rate
*L. anisa*		Pleural infection	
*L. bozemanii*	1 and 2	Pneumonia	
*L. cardiaca*		Native endocarditis	
*L. cincinnatiensis*		Pneumonia	
*L. clemsonensis*		Pneumonia	
*L. dumoffii*		Legionnaires’ disease	
*Legionella feeleii*	1 and 2	Pontiac fever	
*L. hackeliae*	1 and 2	Pneumonia	
*L. jordanis*		Endocarditis	
*L. lansingensis*		Pneumonia	
*Legionella longbeachae*	1 and 2	Pneumonia	
*L. maceachernii*		Pneumonia	
*L. micdadei*		Opportunistic pneumonia	
*L. parisiensis*		Pneumonia	
*L. pneumophila*	1–15	Pontiac fever, Legionnaires’ disease, and pneumonia	7–25%

## Data Availability

Not applicable.
